# Prevalence and determinants of hypertension among adult population in Nepal: Data from Nepal Demographic and Health Survey 2016

**DOI:** 10.1371/journal.pone.0198028

**Published:** 2018-05-31

**Authors:** Mehedi Hasan, Ipsita Sutradhar, Tahmina Akter, Rajat Das Gupta, Hemraj Joshi, Mohammad Rifat Haider, Malabika Sarker

**Affiliations:** 1 BRAC James P. Grant School of Public Health, BRAC University, Dhaka, Bangladesh; 2 Institute of Statistical Research and Training, University of Dhaka, Dhaka, Bangladesh; 3 Child Health Program Office, Lifeline Nepal, Kathmandu, Nepal; 4 Department of Health Services Policy and Management, Arnold School of Public Health, University of South Carolina, Columbia, South Carolina, United States of America; Shanghai Institute of Hypertension, CHINA

## Abstract

Like other developing countries, Nepal is currently going through epidemiological transition along with rising burden of Non-communicable Diseases. However, since 2013, no study investigated the prevalence and determinants of hypertension in Nepal involving nationally representative sample. Therefore, this study aimed to find out the current prevalence of hypertension in Nepal and its determinants using the latest nationally representative data obtained from Nepal Demographic and Health Survey (NDHS) 2016. The NDHS 2016 collected data on hypertension from 13,304 men and women aged 18 years and above from 5,520 urban and 5,970 rural households covering seven administrative provinces and three ecological zones. Participants were considered as hypertensive when their systolic blood pressure was ≥140 mmHg and/or diastolic blood pressure was ≥90 mmHg and/or they reported taking antihypertensive medication. A total of 19.9% study participants were diagnosed as hypertensive of which majority were male (male-24.3%, female-16.9%), ever married (ever married-21.7%, unmarried-6.1%) and residents of urban area (urban-20.9%, rural-18.3%). Hypertension prevalence has shown growing trend with the increase of age. This prevalence was also higher among rich and overweight/obese individuals. In multivariable logistic regression analysis, older age, male gender, better education, residence at urban area and province 4 and 5 and being overweight/obese were found positive association with having hypertension. When the determinants of hypertension were stratified by sex of the participants, difference was observed in case of age group, education and place of residence. As one out of every five individuals in Nepal are hypertensive, public health initiatives are immediately required for prevention and control of hypertension to reduce mortality and morbidity associated with this progressive disease.

## Introduction

Hypertension is a form of cardiovascular disorder that results from a wide range of interconnected etiologies [1]. Untreated and uncontrolled hypertension leads to structural and functional abnormalities of cardiovascular system, which ultimately harm the vital organs of body, e.g., heart, kidneys, brain [2]. Henceforth, hypertension remains one of the foremost causes of death and disability all over the world [3]. In 2001, globally 13·5% of total premature deaths and 6·0% of total Disability Adjusted Life Year (DALY) were caused by hypertension [4]. In addition, 9.4 million people expire every year due to hypertension related complications [5]. South Asia is the home to almost 25% of total world’s population and Non-communicable diseases (NCDs) are accountable for nearly half of the disease burden in this region [6]. Hypertension and related complications are major contributors to death and disability in South Asian countries like India, Bangladesh, Nepal, Bhutan and Sri Lanka [7].

Nepal is currently going through epidemiological transition along with rising burden of NCDs [8]. Different studies have found that prevalence of hypertension among Nepalese population is between 21% and 34% [9–14]. Two cross-sectional studies conducted in a rural area of the Kathmandu valley have found that the burden of hypertension increased almost threefold within 25 years period of time from 1981 to 2006 among individuals aged 21 years and above [11]. In Nepal, the World Health Organization (WHO) implemented ‘STEP wise approach to surveillance’ (STEPS) using nationally representative sample in 2008 and 2013 to identify the prevalence and risk factors of major NCDs. These surveys also found that hypertension prevalence among 15–69 years Nepalese population rose from 21.5% in 2008 to 26.0% in 2013 [1, 2]. However, since 2013, no study has been carried out in Nepal involving nationally representative sample that might give information about current magnitude of this disease. In addition, previous studies excluded population who were more than 70 years old, which impedes to get complete idea about the burden of hypertension in Nepal. Therefore, this study aimed to find out the current prevalence of hypertension in Nepal along with its determinants among adult Nepalese population (aged ≥ 18 years) using the latest nationally representative data obtained from Nepal Demographic and Health Survey (NDHS) 2016. Findings of our study will offer a strong insight to the pertinent stakeholders on the current scenario of hypertension in Nepal, which eventually will help them to set target specific intervention for different group of people on priority basis for prevention and control of this progressive disease.

## Materials and methods

### Study design

Nepal Demographic and Health Survey (NDHS) 2016 data was used to conduct this study, which is a nationally representative survey. Measure Evaluation carries out and manages the survey all over the world, however, in Nepal it was conducted by NEW ERA under the supervision of the Ministry of Health, Nepal (MOH). The NDHS 2016 was conducted in last half of 2016 and used revised version of sampling frame of National Population and Housing Census (NPHC). The sampling frame was revised because in Nepal, urban/rural classification has been changed at the ward level; some new places have been declared as municipalities and some municipalities have been reformed in 2015. This change also divided Nepal into seven provinces (province 1, province 2, province 3, province 4, province 5, province 6, and province 7). Every province was split into urban and rural areas and comprises several districts. Rural areas and urban areas were then subdivided into wards, however, urban areas were further divided into enumeration area (EA) because of having more household than rural areas. Wards were considered as primary sampling unit (PSU) for both urban areas and rural areas [15].

The NDHS 2016 used two stage stratified cluster sample of households and the stratification was achieved based on urban and rural settings. In the first stage of sampling, PSUs were nominated by probability proportional to size followed by systematic selection of households from individual PSU during second stage of sampling. However, in urban settings, three stage stratified cluster sample technique was used for household selection. In the first stage, PSUs were chosen by probability proportional to size. However, in second stage, EAs were randomly selected from PSUs followed by systematic selection of households during third stage of sampling. Primarily, 383 wards were selected, of which 184 wards were from urban settings and 199 were from rural settings. From all the selected wards, total 11,490 households (urban-5,520 households and rural- 5,970 households) were chosen for survey. The full NDHS report was published earlier [15].

### Study participants

**Fig 1:**
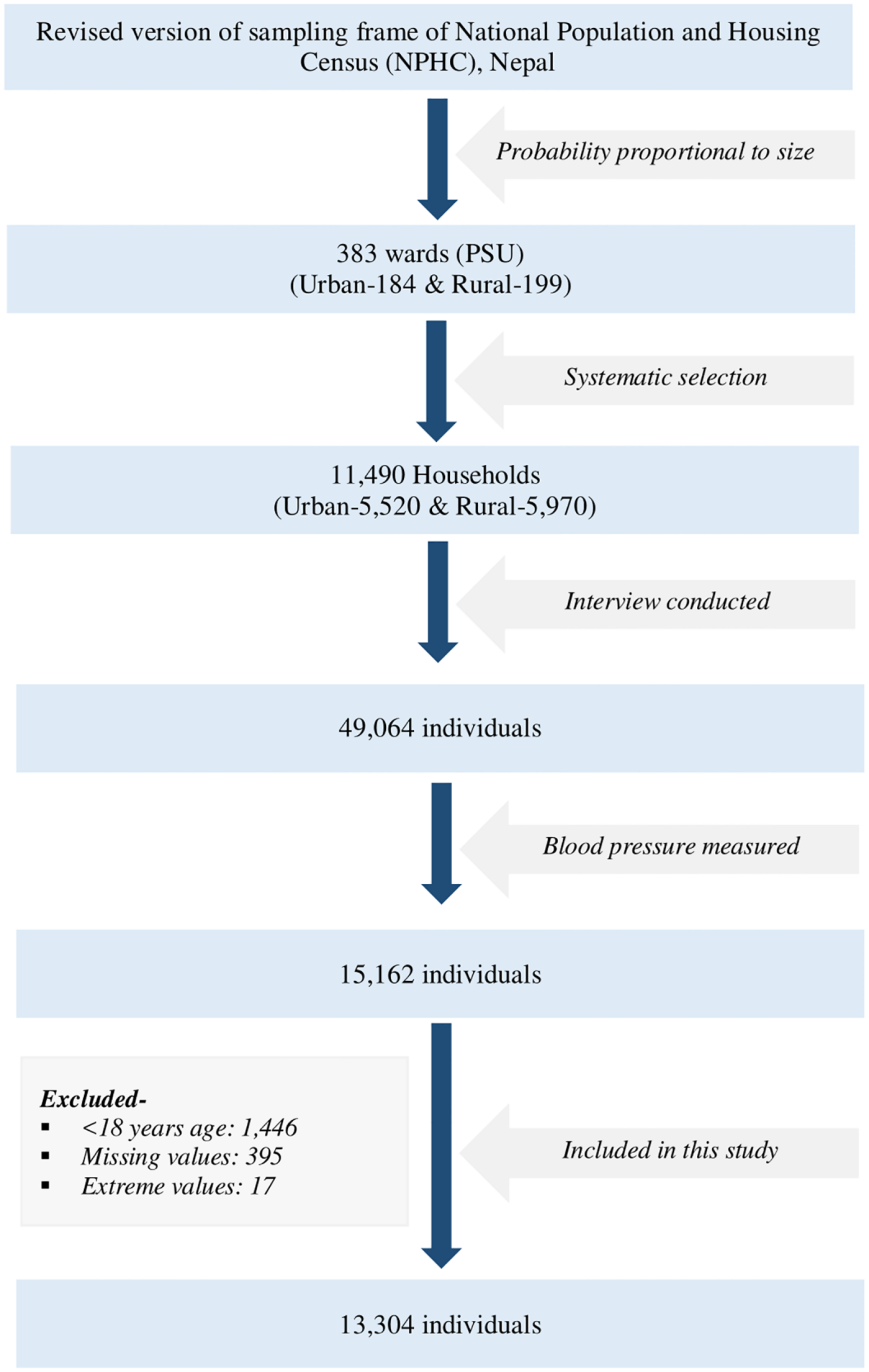
Process of selection of participants.

In NDHS (2016), 49,064 individuals were interviewed, however, BP was measured among 15,162 participants. In our study, we included 13,304 men and women aged 18 years and above whose blood pressure was measured after excluding respondents aged <18 years (n = 1,446), missing values (n = 395) and extreme values (n = 17) (Fig 1).

### Outcome of interest

Hypertension was considered as the outcome variable of this study. Blood pressure was measured three times for individual participant by UA-767F/FAC (A&D Medical) blood pressure monitor. In this survey, first measurement was discarded and then average of second and third measurements was recorded to identify whether the participant was hypertensive or not. A participant having systolic blood pressure ≥140 mmHg and/or diastolic blood pressure ≥90 mmHg was considered as hypertensive [16, 17]. Additionally, Participants taking antihypertensive medication irrespective of their blood pressure during the survey was considered as hypertensive. On the other hand, participants having systolic blood pressure ≥120 mmHg but <140 mmHg and/or diastolic blood pressure ≥80 mmHg but <90 mmHg was considered as pre-hypertensive. For this paper, pre-hypertensive and normotensive were combined as non-hypertensive to make the variable dichotomous.

### Determinants

Age, sex, marital status, education, wealth index, working status, ecological zone, province of residence, place of residence, body mass index, drinking alcohol and caffeine use were considered as determinants of hypertension in this study. Wealth index was segregated into five groups (poorest, poorer, middle, richer, and richest) and was calculated by principal component analysis [15]. Ecologically, Nepal was divided into Mountain, Hill, and the Terai. Moreover, administratively Nepal was divided into seven provinces in 2015 [15]. The body mass index (BMI) was categorized into underweight (<18.5 kg/m^2^), normal (18.5 to 24.9 kg/m^2^), overweight (25.0 to 29.9 kg/m^2^) and obese (≥30.0 kg/m^2^).

### Statistical analysis

All the statistical analyses were performed using Stata 13.0. Univariate analysis of selected variables was performed and presented in terms of frequency. Later, bivariate analysis (cross tabulation) between dependent variable (hypertension) and individual covariate was carried out followed by chi-square test to see the proportional difference between them. Multivariable logistic regression was done to find out the determinants of hypertension. Moreover, multivariable logistic regression was run separately for both male and female participants to see whether the risk factors differ between male and female participants. Both crude and adjusted odds ratio were calculated for each covariate at 95% level of confidence. A determinant was considered significant with p value < .05. Sample weight calculated for NDHS 2016 was used for this paper.

### Ethical consideration

The ethical clearance for NDHS 2016 was taken from Nepal Research council and ICF Macro Institutional Review Board in Calverton, Maryland, USA. Informed written consent was obtained from each respondent prior to the interview.

## Results

A total of 13,304 participants were included in this study of which majority were female (58.1%), ever married (88.3%) and from urban settings (61.1%) (Table 1). More than one-third of our study participants received no education (41.0%). Nearly half of the participants were residents of ecological zone Terai (49.6%) and just over one-fifth were from Province 3 (21.9%). The median age (±SD) of the study participants was 38 (±16.7) years. Highest proportion of these population were from 25–34 years age group (23.2%) followed by 18–24 years age group (20.6%). Most of the study participants (62.0%) had normal BMI, however, 16.7% were underweight and 21.4% were overweight or obese. The overall mean systolic BP (±SD) of the participants was 116.31 (±19.36) mmHg and the mean diastolic BP (±SD) was 77.89 (±11.59) mmHg.

**Table 1:** Characteristic of the study participants by hypertension status (weighted) and prevalence of hypertension, Nepal Demographic and Health Survey (NDHS), 2016 (N = 13,304).

	Total		No Hypertension		Hypertension		Prevalence of Hypertension	p-value
Overall	13,304	-	10,649	80.1	2,655	19.1	19.9	
Variables	n	%	n	%	n	%	%	
**Age Group (in Years)**								<0.001
18–24	2,768	20.6	2,648	24.7	120	4.3	4.3	
25–34	3,030	23.2	2,714	26.1	316	11.8	10.4	
35–44	2,486	18.8	1,970	18.6	516	19.9	20.8	
45–54	2,029	14.9	1,463	13.3	566	21.5	27.9	
55–64	1,601	11.8	1,043	9.6	558	20.6	34.9	
≥65	1,390	10.7	811	7.8	579	21.9	41.7	
**Sex**								<0.001
Male	5,499	42.0	4,164	39.8	1,335	50.4	24.3	
Female	7,805	58.1	6,485	60.2	1,320	49.6	16.9	
**Marital Status**								<0.001
Unmarried	1,481	11.7	1,388	13.82	93	3.6	6.3	
Ever married	11,823	88.3	9,261	86.18	2,562	96.4	21.7	
**Education**								<0.001
No education	5,515	41.0	4,206	39.2	1,309	48.1	23.7	
Primary education	2,281	17.2	1,791	16.9	490	18.2	21.5	
Secondary education	3,676	27.4	3,085	28.6	591	23.0	16.1	
Higher education	1,832	14.4	1,567	15.3	265	10.8	14.5	
**Wealth index**								<0.001
Poor	5,600	37.3	4,553	37.9	1,047	35.2	18.7	
Middle	2,653	20.0	2,179	20.7	474	17.2	17.9	
Rich	5,051	42.7	3,917	41.4	1,134	47.7	22.5	
**Place of residence**								<0.001
Urban	8,395	61.1	6,639	60.1	1,756	65.0	20.9	
Rural	4,909	38.9	4,010	39.9	899	35.0	18.3	
**Ecological zone**								<0.001
Mountain	994	6.4	833	6.6	161	5.6	16.2	
Hill	6,062	44.0	4,695	42.4	1,367	50.3	22.6	
The Terai	6,248	49.6	5,121	51.0	1,127	44.2	18.0	
**Province of Residence**								<0.001
Province 1	1,989	17.6	1,608	17.8	381	16.7	19.2	
Province 2	2,230	20.6	1,866	21.8	364	15.9	16.3	
Province 3	1,904	21.9	1,445	20.8	459	25.9	24.1	
Province 4	1,785	10.3	1,294	9.4	491	14.1	27.5	
Province 5	1,991	16.3	1,548	15.9	443	17.8	22.3	
Province 6	1,619	5.0	1,351	5.3	268	3.9	16.6	
Province 7	1,786	8.3	1,537	8.9	249	5.8	13.9	
**Body Mass Index (BMI)**								<0.001
Underweight	2,207	16.7	1,920	18.1	287	11.0	13.0	
Normal	8,470	62.0	7,036	64.4	1,434	52.4	16.9	
Overweight and Obese	2,627	21.4	1,693	17.5	934	36.6	35.6	
**Drinking alcohol**								0.001
No	13,080	98.41	10,497	98.61	2,583	97.6	19.7	
Yes	224	1.59	152	1.39	72	2.4	32.1	
**Caffeine use**								<0.001
No	12,273	92.12	9,935	93.1	2,338	88.3	19.0	
Yes	1,031	7.88	714	6.9	317	11.7	30.7	

A total of 19.9% of our study participants were identified as hypertensive, however, the prevalence was 31.3% among ≥35 years old respondents. Hypertension prevalence has shown growing trend with the increase of age. Among the study participants aged 65 years or more, the prevalence was highest (41.7%), followed by 55–64 years old (34.9%) (Table 1). Noticeably, one out of every five individuals of 35–44 years age group (20.8%) and one out of every four individuals of 45–54 years age group (27.9%) were hypertensive. This prevalence was significantly higher among male participants (male-24.3%, female-16.9%; *p<0*.*001*); ever married individuals (ever married-21.7%, unmarried-6.3%; *p<0*.*001*) and residents of urban area (urban-20.9%, rural-18.3%; *p<0*.*001*). Higher prevalence of hypertension was also seen among the individuals who received no education (23.7%) or attained only primary education (21.5%) than their counterparts who accomplished secondary education (16.1%) or higher education (14.5%). Prevalence of hypertension was, moreover, greater among participants from rich wealth quintile (22.5%) than the participants from other wealth quintiles. Overweight and obese individuals experienced highest prevalence of hypertension (35.6%) than the individuals having normal (16.9%) or low (13.0%) BMI. This prevalence was also higher among respondents who used to drink alcohol (alcohol-32.1%, no alcohol-19.7%; *p = 0*.*001*) and caffeine (caffeine-30.7%, no caffeine-19.0%; *p<0*.*001*). In Nepal, the prevalence of hypertension shown strong geographic trends, with the highest burden in hill area (22.6%) and in Province 4 (27.5%).

Among the hypertensive patients, only 23% were taking medications. Rest of them were newly diagnosed.

Table 2 demonstrates the logistic regression analysis with Crude Odds Ratios (COR) and Adjusted Odds Ratio (AOR) at 95% level of confidence. In the final model, respondents’ age, sex, education, BMI, place of residence, and province of residence were found significantly associated with the status of hypertension. From multivariable logistic regression analysis, it was revealed that participants having 65 or more years of age and 55–64 years of age were respectively nineteen times (AOR = 19.752, 95% CI: 14.737–26.472, *p*<0.001) and twelve times (AOR = 12.773, 95% CI: 9.625–16.951, *p<0*.*001*) more likely to be hypertensive compared to those who were 18–24 years old. Female gender was found negatively associated with developing hypertension after adjusting for potential confounders (AOR = 0.742, 95% CI: 0.658–0.836, *p<0*.*001*). Education, additionally, was found to be associated with status of hypertension among our study participants. In our study, respondents who attained primary education and secondary education were 24% (AOR = 1.239, 95% CI: 1.056–1.453, *p = 0*.*009*) and 27% (AOR = 1.268, 95% CI: 1.072–1.499, *p = 0*.*006*) more likely to be hypertensive than those who had no education. Noticeably, there was no significant association with higher education and hypertension (AOR = 1.246, 95% CI: 0.994–1.562, *p = 0*.*057*). Residence in province 4 and 5 were also found positively associated with developing hypertension among our study participants however, negative association was found with residence in rural settings (AOR = 0.882, 95% CI: 0.788–0.988, *p = 0*.*030*). Drinking alcohol or using caffeine had no significant association with the risk of having hypertension in our study.

**Table 2:** Logistic regression analysis of determinants of hypertension among adults in Nepal, Nepal Demographic and Health Survey (NDHS), 2016 (N = 13,304).

Variables	COR*	CI**	p-value	AOR***	CI**	*p*-value
**Age Group (in Years)**						
18–24	Ref.			Ref.		
25–34	2.623	2.059–3.341	<0.001	2.101	1.604–2.752	<0.001
35–44	6.199	4.93–7.795	<0.001	4.852	3.708–6.348	<0.001
45–54	9.393	7.472–11.809	<0.001	8.337	6.329–10.981	<0.001
55–64	12.485	9.915–15.722	<0.001	12.773	9.625–16.951	<0.001
≥65	16.171	12.813–20.408	<0.001	19.752	14.737–26.472	<0.001
**Sex**						
Male	Ref.			Ref.		
Female	0.650	0.588–0.718	<0.001	0.742	0.658–0.836	<0.001
**Marital Status**						
Unmarried						
Ever married	4.340	3.435–5.484	<0.001	1.112	0.835–1.481	0.466
**Education**						
No education	Ref.			Ref.		
Primary education	0.878	0.766–1.006	0.061	1.239	1.056–1.453	0.009
Secondary education	0.657	0.579–0.745	<0.001	1.268	1.072–1.499	0.006
Higher education	0.574	0.482–0.683	<0.001	1.246	0.994–1.562	0.057
**Wealth index**						
Poor	Ref.			Ref.		
Middle	0.892	0.781–1.018	0.091	0.973	0.829–1.141	0.733
Rich	1.241	1.113–1.383	<0.001	1.054	0.908–1.224	0.486
**Place of residence**						
Urban	Ref.			Ref.		
Rural	0.813	0.736–0.899	<0.001	0.882	0.788–0.988	0.030
**Ecological zone**						
Mountain	Ref.			Ref.		
Hill	1.410	1.157–1.717	0.001	1.106	0.885–1.381	0.375
The Terai	1.030	0.847–1.252	0.768	0.957	0.742–1.235	0.736
**Province of Residence**						
Province 1	Ref.			Ref.		
Province 2	0.777	0.658–0.918	0.003	0.990	0.816–1.201	0.920
Province 3	1.330	1.12–1.579	0.001	1.178	0.966–1.435	0.105
Province 4	1.603	1.369–1.878	<0.001	1.410	1.17–1.699	<0.001
Province 5	1.195	1.019–1.4	0.028	1.382	1.159–1.649	<0.001
Province 6	0.782	0.65–0.941	0.009	0.990	0.797–1.23	0.929
Province 7	0.695	0.579–0.834	<0.001	0.877	0.718–1.07	0.195
**Body Mass Index (BMI)**						
Underweight	Ref.			Ref.		
Normal	1.333	1.136–1.563	<0.001	1.612	1.355–1.916	<0.001
Overweight and Obese	3.436	2.888–4.089	<0.001	3.999	3.264–4.900	<0.001
**Drinking alcohol**						
No	Ref.			Ref.		
Yes	1.738	1.268–2.382	0.001	1.063	0.756–1.495	0.726
**Caffeine use**						
No	Ref.			Ref.		
Yes	1.789	1.52–2.105	<0.001	1.178	0.984–1.411	0.074

*COR: Crude Odds Ratio.

**CI: Confidence Interval.

***AOR: Adjusted Odds Ratio.

When the determinants of hypertension were stratified by sex of the participants, difference was observed in case of age group, education and place of residence (Table 3). For instance, female of 35–44 years and 45–54 years age groups were five times (AOR = 5.032, 95% CI: 3.539–7.155, *p<0*.*001*) and nine times (AOR = 9.038, 95% CI: 6.272–13.023, *p<0*.*001*) more likely to develop hypertension respectively than the female of 18–24 years age group. However, the risk of developing hypertension was four times (AOR = 4.344, 95% CI: 2.815–6.705, *p<0*.*001*) and seven times (AOR = 7.203, 95% CI: 4.638–11.186, *p<0*.*001*) higher for 35–44 years and 45–54 years age male than the male of 18–24 years age group. Similarly, female of 55–64 years and ≥65 years age groups were fifteen times (AOR = 15.340, 95% CI: 10.488–22.436, *p<0*.*001*) and twenty-six times (AOR = 26.408, 95% CI: 17.805–39.167, *p<0*.*001*) more likely to develop hypertension respectively, though, the risk of developing hypertension was ten times (AOR = 10.179, 95% CI: 6.531–15.865, *p<0*.*001*) and fifteen times (AOR = 15.112, 95% CI: 9.611–23.763, *p<0*.*001*) higher for 35–44 years and 45–54 years age male respectively. Among male participants of our study who lived in the rural area had 22% lower risk of being hypertensive (AOR = 0.788, 95% CI: 0.671–0.925, *p = 0*.*004*) than the urban residents, however, no such association was found in females. Additionally, men receiving education were more likely to have hypertension than their counterparts having no education (primary- AOR = 1.452, 95% CI: 1.165–1.811, *p = 0*.*001*; secondary- AOR = 1.497, 95% CI: 1.186–1.889, *p = 0*.*001*; higher- AOR = 1.396, 95% CI: 1.186–1.889, *p = 0*.*029*), nevertheless, no such association was found in case of females. Moreover, men from Hills zone showed 43% more risk of being hypertensive than the male residents from the mountain region (AOR = 1.434, 95% CI: 1.045–1.968, *p = 0*.*025*), though, among female respondents, no association was found between status of hypertension and ecological zone. Overweight and obesity as well as residing in province 4 and 5 were found as significant predictors of hypertension among both male and female participants of our study.

**Table 3:** Logistic regression analysis of determinants of hypertension among adults in Nepal stratified by sex, Nepal Demographic and Health Survey (NDHS), 2016 (N = 13,304).

Variables	Female (n = 7,805)						Male (n = 5,499)					
	COR	CI	p-value	AOR	CI	p-value	COR	CI	p-value	AOR	CI	p-value
**Age Group (in Years)**												
18–24	Ref.			Ref.			Ref.			Ref.		
25–34	2.194	1.538–3.13	<0.001	1.611	1.112–2.334	0.012	3.330	2.378–4.663	<0.001	2.706	1.778–4.119	<0.001
35–44	6.877	4.99–9.477	<0.001	5.032	3.539–7.155	<0.001	5.581	4.015–7.757	<0.001	4.344	2.815–6.705	<0.001
45–54	10.915	7.926–15.03	<0.001	9.038	6.272–13.023	<0.001	7.822	5.632–10.864	<0.001	7.203	4.638–11.186	<0.001
55–64	15.892	11.495–21.97	<0.001	15.340	10.488–22.436	<0.001	9.365	6.738–13.017	<0.001	10.179	6.531–15.865	<0.001
≥65	22.453	16.187–31.144	<0.001	26.408	17.805–39.167	<0.001	11.117	7.973–15.501	<0.001	15.112	9.611–23.763	<0.001
**Marital Status**												
Unmarried	Ref.			Ref.			Ref.			Ref.		
Ever married	5.563	3.619–8.552	<0.001	1.436	0.902–2.284	0.127	4.394	3.31–5.833	<0.001	1.090	0.729–1.632	0.674
**Education**												
No education	Ref.			Ref.			Ref.			Ref.		
Primary education	0.597	0.485–0.736	<0.001	1.189	0.923–1.532	0.180	1.053	0.862–1.286	0.615	1.452	1.165–1.811	0.001
Secondary education	0.384	0.315–0.469	<0.001	1.129	0.866–1.473	0.369	0.833	0.692–1.003	0.054	1.497	1.186–1.889	0.001
Higher education	0.348	0.252–0.481	<0.001	1.228	0.832–1.811	0.301	0.717	0.569–0.903	0.005	1.396	1.035–1.883	0.029
**Wealth index**												
Poor	Ref.			Ref.			Ref.			Ref.		
Middle	0.922	0.77–1.105	0.380	0.999	0.8–1.247	0.993	0.860	0.706–1.048	0.135	0.932	0.74–1.174	0.552
Rich	1.285	1.106–1.493	0.001	1.094	0.886–1.35	0.405	1.167	0.997–1.367	0.055	0.991	0.802–1.225	0.935
**Place of residence**												
Urban	Ref.			Ref.			Ref.			Ref.		
Rural	0.878	0.765–1.007	0.063	0.989	0.844–1.159	0.892	0.751	0.648–0.869	<0.001	0.788	0.671–0.925	0.004
**Ecological zone**												
Mountain	Ref.			Ref.			Ref.			Ref.		
Hill	1.184	0.905–1.549	0.218	0.863	0.631–1.181	0.357	1.741	1.301–2.33	<0.001	1.434	1.045–1.968	0.025
The Terai	0.933	0.715–1.218	0.611	0.833	0.583–1.192	0.317	1.158	0.869–1.543	0.318	1.134	0.788–1.632	0.498
**Province of Residence**												
Province 1	Ref.			Ref.			Ref.			Ref.		
Province 2	0.723	0.575–0.91	0.006	1.029	0.784–1.35	0.837	0.835	0.657–1.063	0.144	0.973	0.739–1.282	0.847
Province 3	1.178	0.93–1.491	0.174	1.050	0.793–1.391	0.731	1.501	1.166–1.93	0.002	1.334	1.006–1.77	0.045
Province 4	1.497	1.207–1.856	0.000	1.417	1.091–1.841	0.009	1.773	1.401–2.243	<0.001	1.442	1.1–1.891	0.008
Province 5	1.130	0.911–1.403	0.266	1.346	1.055–1.716	0.017	1.287	1.017–1.628	0.036	1.421	1.096–1.842	0.008
Province 6	0.568	0.434–0.743	<0.001	0.794	0.579–1.088	0.151	1.070	0.825–1.388	0.608	1.237	0.913–1.677	0.169
Province 7	0.556	0.43–0.719	<0.001	0.685	0.513–0.915	0.010	0.911	0.701–1.184	0.486	1.087	0.82–1.441	0.562
**Body Mass Index (BMI)**												
Underweight	Ref.			Ref.			Ref.			Ref.		
Normal	1.225	0.995–1.508	0.056	1.787	1.416–2.254	<0.001	1.382	1.079–1.771	0.011	1.519	1.174–1.965	0.001
Overweight and Obese	3.217	2.574–4.02	<0.001	4.381	3.339–5.748	<0.001	4.044	3.069–5.33	<0.001	3.950	2.908–5.364	<0.001
**Drinking alcohol**	Ref.			Ref.			Ref.			Ref.		
No												
Yes	2.414	1.317–4.422	0.004	1.130	0.568–2.247	0.728	1.308	0.903–1.895	0.156	1.075	0.73–1.583	0.714
**Caffeine use**												
No	Ref.			Ref.			Ref.			Ref.		
Yes	1.947	1.526–2.484	<0.001	1.146	0.871–1.508	0.330	1.525	1.224–1.900	<0.001	1.191	0.939–1.512	0.150

## Discussion

### Prevalence of hypertension

To the best of our knowledge, this was the first reported study that investigated the prevalence and determinants of hypertension among Nepalese population using the latest nationally representative data obtained from NDHS 2016. Finding of our study reveals that about one-fifth (19.9%) of Nepalese adults were hypertensive. Prevalence was higher among older age group, ever married, rich and male participants; participants having no education, residing in urban area, hill zone and province 4; participants drinking alcohol, using caffeine and with higher BMI. In our study, older age, male gender, receiving education, urban residence, living in province 4 and 5 as well as being overweight or obese were found to have positive association with being hypertensive.

In our study, the overall prevalence of hypertension was found as 19.9%. This prevalence is lower than the estimates found in 2013 WHO STEPS survey conducted among 15–69 years old Nepalese people (25.7%) [14]. This difference might be due to dissimilarity in survey design, age of the participants and other methodological issues. Prevalence of hypertension was found 29.6% in our study while measured for ≥35 years old respondents. This prevalence is higher than the prevalence of hypertension in Bangladesh among similar age group participants but lower than that of Pakistan. According to the Bangladesh Demographic and Health Survey (2011), 24.4% adults aged 35 years or older were hypertensive in Bangladesh [18]. On the other hand, in Pakistan, 42.3% 35 to 74 years of age had hypertension [19]. As one out of every five adults is hypertensive, it can be undoubtedly stated that a huge burden of hypertension is currently prevailing in Nepal. Untreated and uncontrolled high blood pressure is the reason for developing a wide range of complications e.g. ischemic heart disease, pulmonary hypertension, heart failure, renal disease, conduction disorders and cerebrovascular diseases like stroke [20]. It is also attributable to premature deaths and disability [20]. In addition, hypertension driven complications uplift the expense of medical care [21]. Therefore, it is a timely need for policy makers of the country to offer prevention and control of hypertension the highest priority considering its health and economic burden.

### Determinants of hypertension

It was revealed from our study that, the risk of developing hypertension upturned with increasing age and the risk was almost 20-fold higher (AOR = 19.752) for respondents aged 65 years or more. Age is a non-modifiable factor of hypertension [22]. As Nepal is currently confronting demographic transition due to declining fertility rate and improved life expectancy, the rise of number of elderly group of people is imminent in upcoming days [23]. This incident has the potential to upsurge the number of hypertensive patients in Nepal and therefore the burden of this disease seems to intensify in forthcoming times [24]. Male gender was also found to have higher risk of developing hypertension compared to their female counterparts in our study. This gender difference in case of developing hypertension has been well documented in previous researches conducted in Nepal [11,14,25] and in other countries [14,26–28].

In our study, we found that education has a positive relationship with the risk of being hypertensive. This finding is supported by previous studies conducted in six middle income countries [29] and in Bangladesh [30] where it was reported that prevalence of hypertension was greater among individuals who received higher education. Sedentary life style and intake of high calorie diet by the population belonging to higher educational status might explain this pattern [31–33]. However, we did not find positive association between higher socio economic status with the risk of experiencing hypertension which was exhibited in other studies [30,31]. Moreover, the urban residents of Nepal had higher odds of having hypertension, which is coherent with the findings of previous researches conducted in different geographical and cultural context across the continents e.g. Nepal, Bangladesh, Uganda and Kenya [14,30,34–36]. Sedentary lifestyles coupled with junk food consumption, which has been emerged as an inevitable consequence of urbanization, were reported as the foremost reasons of this in those studies [34,37,38]. Overweight and obesity was emerged as a significant factor associated with hypertension in our study. This relationship was also shown in previous studies in different parts of the world [28,29–32,39–41]. Noticeably, WHO STEPS survey 2013 has reported that prevalence of excessive BMI has been growing in both urban and rural areas of Nepal, whereas, the level of physical activity was very low among these people [14]. As Nepal is confronting nutritional transition, upward trend of the overweight and obesity is expected to persist in upcoming years that, in turn, can expand the burden of hypertension in Nepal.

### Recommendations

Considering the emerging demographic, nutritional and epidemiological transition, stakeholders of Nepal should keep focus on prevention and control of hypertension. Recently, NCDs have been admitted as an important public health issue in the National Health Policies and National Health Sector Plans of Nepal [42]. For instance, NCD related services are being offered using the existing health system [43]. However, current NCD platforms are mostly focused on health promotion activities using Behavior Change Communication (BCC) [43]. NCDs are not part of the essential health care service package (ESP) and health care providers are not well trained, therefore, majority of the people do not have access to good quality of care for NCDs like hypertension and diabetes [44]. In addition, antihypertensive drug is not included in ESP, hence, availability of essential medications at no or low cost is still not the reality for Nepalese people [44]. Therefore, it is a timely need for pertinent stakeholders of Nepal to introduce a novel approach to generate financially sustainable and target specific programs for early diagnosis, treatment and control of hypertension through a group of well-trained health care providers. Offering antihypertensive drugs at no or low cost and ensuring enabling environment for lifestyle modification is also needed in this regard. High-risk group of population ought to be given priority while designing community-based programs for screening, diagnosis and management of hypertension. Further research is additionally warranted in order to identify implementation challenges of current programs and to imply this knowledge in future. Additionally, strong public private partnership involving different government and non-government organizations should work together to combat the current burden of hypertension in Nepal.

### Strengths and limitations

As this study utilized a nationally representative sample from Nepal, the results can be generalized to the target population (≥18 years old population in Nepal). Moreover, due to the utilization of standard and valid tools for data collection by NDHS, the probability of existence of measurement error is less in our study in comparison to other single cross sectional studies conducted in Nepal. However, this study has some potential limitations. We could not measure the associations between hypertension and several important determinants like dietary habits, physical activity, diabetes, salt intake, smoking and family history of hypertension due to absence of the data in the survey.

## Conclusions

Non-communicable diseases are now a global concern, as they not only cause premature death but also impose huge health and economic burden on a nation. Our study indicates that, in Nepal, one out of every five individuals aged 18 years or above is hypertensive. Older age, male gender, receiving education and being overweight or obese were found positive association with being hypertensive. People residing in urban area and in province 4 and 5 are also found at higher risk to experience hypertension. Public health efforts are urgently warranted for effective prevention and control of this progressive disease for diverse group of people from different geographic area on priority basis to reduce its health and economic burden.
